# MntP and YiiP Contribute to Manganese Efflux in Salmonella enterica Serovar Typhimurium under Conditions of Manganese Overload and Nitrosative Stress

**DOI:** 10.1128/spectrum.01316-21

**Published:** 2022-01-12

**Authors:** Annie Ouyang, Kendall M. Gasner, Stephanie L. Neville, Christopher A. McDevitt, Elaine R. Frawley

**Affiliations:** a Rhodes College Biology Department, Memphis, Tennessee, USA; b Department of Microbiology and Immunology, The Peter Doherty Institute for Infection and Immunity, The University of Melbourne, Victoria, Australia; Indian Institute of Science Bangalore

**Keywords:** *Salmonella*, efflux pumps, manganese, metal ion homeostasis, nitric oxide

## Abstract

The divalent transition metal cation manganese is important for protein function, particularly under conditions of iron limitation, nitrosative stress, and oxidative stress, but can mediate substantial toxicity in excess. Salmonella enterica serovar Typhimurium possesses multiple manganese importers, but the pathways for manganese efflux remain poorly defined. The *S*. Typhimurium ATCC 14028s genome was analyzed for putative manganese export pathways, which identified a previously uncharacterized homologue of the Escherichia coli manganese exporter *mntP*, *stm1834,* and two cation diffusion facilitator family transporters, *zitB* (*stm0758*) and *yiiP* (*stm4061*). Manganese acquisition by *S*. Typhimurium has been shown to occur in response to nitric oxide, an important chemical mediator of the mammalian innate immune response. However, cellular manganese can rapidly return to prechallenge levels, strongly suggesting that one or more *S*. Typhimurium exporters may contribute to this process. Here, we report that *mntP* and *yiiP* contribute to manganese resistance and export in *S*. Typhimurium. YiiP, also known as FieF, has previously been associated with zinc and iron transport, although its physiological role remains ambiguous due to a lack of zinc-sensitive phenotypes in *yiiP* mutant strains of *S*. Typhimurium and E. coli. We report that *S*. Typhimurium Δ*mntP* Δ*yiiP* mutants are exquisitely sensitive to manganese and show that both YiiP and MntP contribute to manganese efflux following nitric oxide exposure.

**IMPORTANCE** Transition metal cations are required for the function of many proteins but can mediate toxicity when present in excess. Identifying transporters that facilitate metal ion export, the conditions under which they are expressed, and the role they play in bacterial physiology is an evolving area of interest for environmental and pathogenic organisms. Determining the native targets of metal transporters has proved challenging since bioinformatic predictions, *in vitro* transport data, and mutant phenotypes do not always agree. This work identifies two transporters that mediate manganese efflux from the Gram-negative pathogen Salmonella enterica serovar Typhimurium in response to manganese overload and nitric oxide stress. While homologues of MntP have been characterized previously, this is the first observation of YiiP contributing to manganese export.

## INTRODUCTION

Metal cofactors are important to the function of many proteins, including nearly half of all enzymes (1, 2). Salmonella enterica serovar Typhimurium (*S*. Typhimurium), like many bacteria, possesses a variety of high-affinity import systems for divalent cations including magnesium, iron, zinc, and manganese (3–9). Import of manganese, which is not required for growth of *S*. Typhimurium under standard laboratory conditions, is important for growth under conditions of oxidative stress, nitrosative stress, and iron limitation, as well as within host environments (8, 10–14).

While manganese is beneficial under a variety of stress conditions, it can be toxic at elevated concentrations. Studies in Escherichia coli have shown that excess intracellular manganese inhibits heme biosynthesis while chronic manganese stress ultimately leads to iron depletion and impaired formation of Fe-S cluster proteins (15, 16). Together, these effects can result in inhibition of energy-generating and biosynthetic pathways. In Bacillus subtilis, which requires manganese for growth, excess manganese has been associated with impaired function of the cytochrome aa_3_ heme-copper menaquinol oxidase (QoxABCD) of the electron transport chain (17). Therefore, while increased cellular manganese may benefit bacteria when challenged with a specific physiological or chemical stress, efflux of manganese may be necessary upon alleviation of the aforementioned stress.

Three types of manganese efflux systems have been identified in prokaryotes to date. Transporters from the cation diffusion facilitator (CDF) family are widely prevalent among prokaryotic species with family members implicated in transport of zinc, cadmium, cobalt, nickel and manganese, depending on the sequence motifs present in metal binding regions (18). The prokaryotic CDF manganese exporter MntE was first identified in Streptococcus pneumoniae and has since been studied in Staphylococcus aureus, Enterococcus faecalis, and Streptococcus pyogenes (19–22). B. subtilis relies on two CDF family transporters for manganese efflux with MneP functioning as the primary exporter and MneS playing a secondary role (23). P_1B_-ATPases have been shown to export a range of transition metal ions, with manganese export first established for CtpC from Mycobacterium tuberculosis (24). MntP, which lacks homology to other established classes of manganese exporters, was first characterized in Xanthomonas oryzae, E. coli, and Neisseria meningitidis (as MntX) (25–27). This architecturally distinct transporter has since been shown to have orthologs in additional species.

Previously, we showed that manganese acquisition by MntH, SitABCD, and ZupT is important for *S*. Typhimurium nitrosative stress resistance. Furthermore, total cellular manganese was restored to prechallenge levels following the resolution of the stress (14). In this study, we sought to identify and characterize *S*. Typhimurium efflux systems that contribute to manganese homeostasis in response to stress. We show that orthologs of the E. coli transporters MntP and YiiP protect *S*. Typhimurium against manganese intoxication and mediate manganese efflux during the late-stage response to nitrosative stress.

## RESULTS

### STM1834 (MntP) protects *S*. Typhimurium against excess manganese.

**FIG 1:**
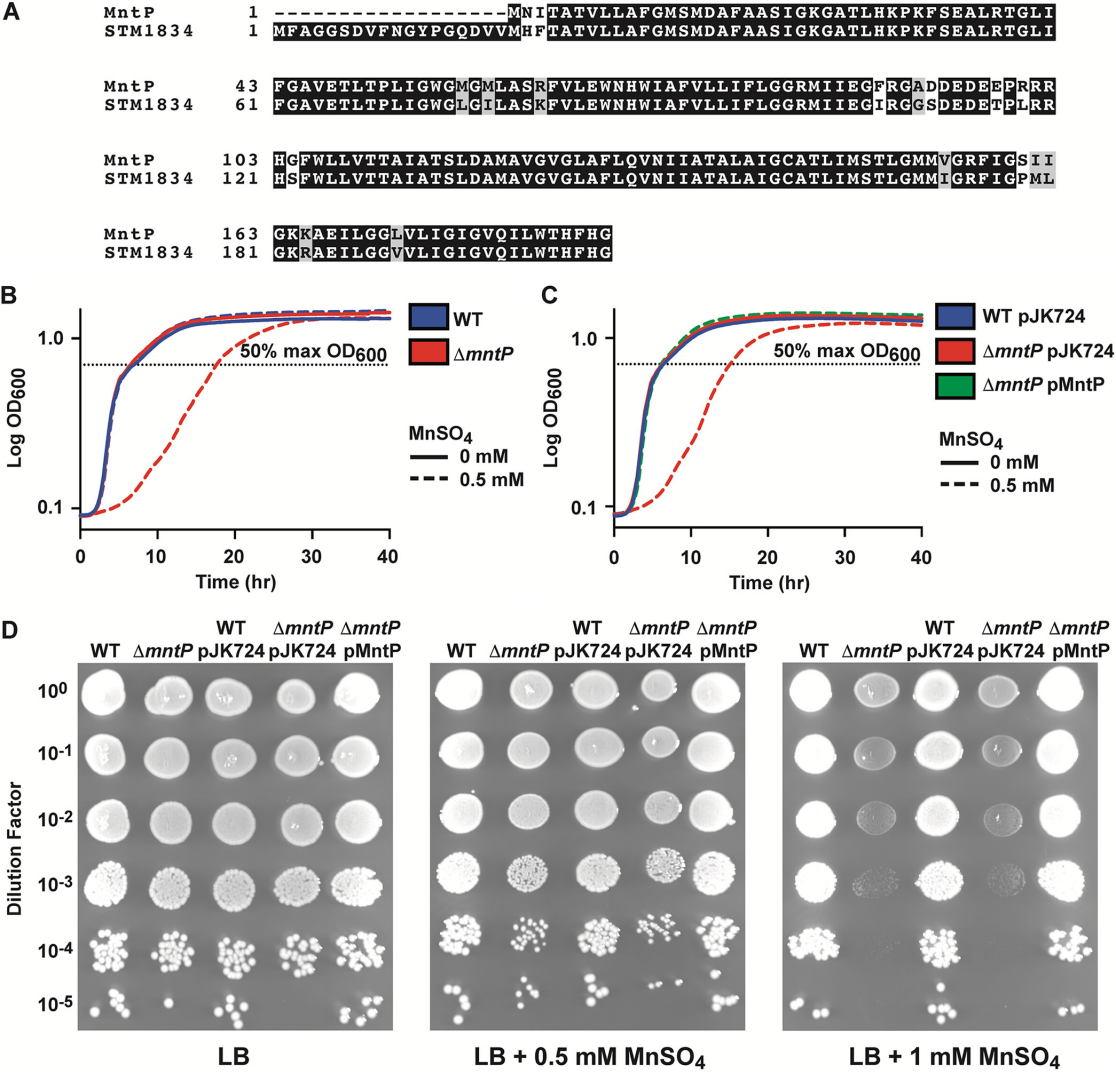
Deletion of the putative efflux transporter *stm1834* (*mntP*) delays growth of *S*. Typhimurium in 0.5 mM manganese. (A) Amino acid sequence alignment of E. coli MG1655 MntP (B3915) with *S*. Typhimurium STM1834. Identical residues are shown in black, while similar residues are shown in gray. (B) Growth phenotypes of *S*. Typhimurium wild-type (WT) and Δ*mntP* strains in LB with or without 0.5 mM MnSO_4_ supplementation. The Δ*mntP* strain was delayed exiting lag phase relative to WT in the presence of 0.5 mM MnSO_4_ (*P* < 0.001). (C) Growth phenotypes of *S*. Typhimurium empty vector strains WT pJK724 and Δ*mntP* pJK724 compared to plasmid-based complementation strain Δ*mntP* pMntP. In 0.5 mM MnSO_4_ the Δ*mntP* pJK724 strain was delayed exiting lag phase compared to both WT pJK724 and Δ*mntP* pMntP (*P* < 0.001). Data for (B) and (C) are the mean of 4 independent experiments. Statistical significance of differences between strains was determined by the time (hr) to reach 50% maximum growth (OD_600_; dashed line) by unpaired two-tailed *t* test. (D) Growth of strains from (B) and (C) assessed using spot assays. Dilutions of OD_600_ = 0.3 cultures were spotted onto LB agar with and without MnSO_4_ supplementation. A representative spot assay for each condition is shown, selected from 3 independent biological replicates.

E. coli and *S*. Typhimurium share similar genetic sequences at many loci, so the *S*. Typhimurium genome was searched for proteins with homology to MntP from E. coli. One match, at locus *stm1834* (Fig. 1A), was identified with 91% identity and 96% similarity (over 188 amino acids) to E. coli MntP. To determine the function of the putative *S*. Typhimurium *mntP* ortholog, a deletion mutant was generated. The mutant was then grown in the presence of 0.5 mM MnSO_4_ and the phenotype compared to the wild-type parental strain. The *S*. Typhimurium Δ*mntP* strain was delayed for growth in excess manganese compared to the wild-type (Fig. 1B). Constitutive expression of *S*. Typhimurium *mntP* from a low-copy-number plasmid (pMntP) complemented the growth defect of a Δ*mntP* mutant (Fig. 1C). Consistent with the growth phenotype data, spot plate assays on manganese supplemented media showed that the *S*. Typhimurium Δ*mntP* strain had decreased growth on 0.5 mM MnSO_4_ and decreased survival on 1 mM MnSO_4_, while expression of *mntP* complemented these phenotypes (Fig. 1D). Taken together, these data are consistent with previous studies of MntP function in E. coli and suggest that *stm1834* encodes a manganese exporter homologous to E. coli MntP (26).

### MntP is not solely responsible for manganese efflux following NO· treatment.

**FIG 2:**
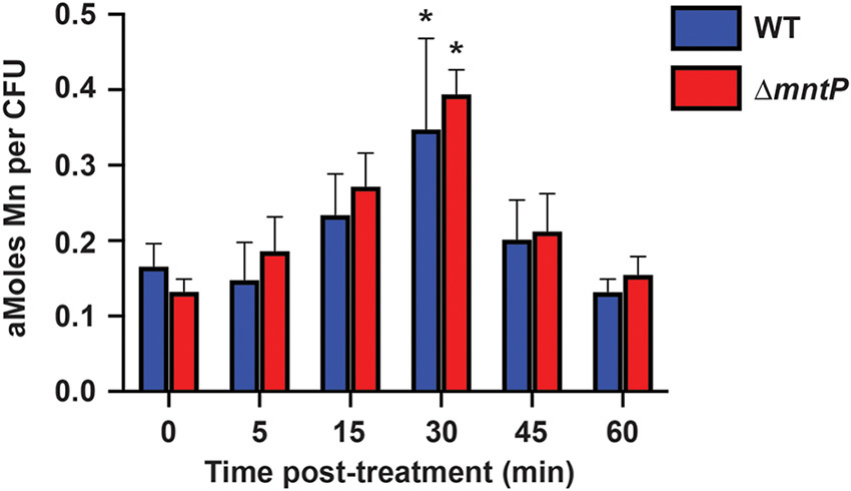
Intracellular manganese levels are not significantly different in Δ*mntP* *S*. Typhimurium compared to the wild-type following NO· treatment. Total cellular manganese in *S*. Typhimurium wild-type (WT) and Δ*mntP* strains determined by ICP-MS following addition of 2 mM DEANO. Manganese levels were significantly elevated (*) for WT (*P* = 0.026) and Δ*mntP* (*P* < 0.001) at 30 min posttreatment compared to 0 min. At no time were manganese levels in the Δ*mntP* strain significantly different from WT. Data are the mean (± standard deviation) of 3 independent experiments. Statistical significance of differences between strains and across time points for each strain was determined by unpaired two-tailed *t* test.

To determine if manganese export by MntP is responsible for returning cellular manganese levels to pretreatment levels following NO· exposure, we compared the phenotypes of wild-type and Δ*mntP S*. Typhimurium by inductively coupled plasma-mass spectrometry (ICP-MS). Here, we used conditions defined in our prior studies of *S*. Typhimurium wherein the application of NO· stress alters metal homeostasis and induces manganese accumulation (14, 28). Accordingly, cultures were treated with 2 mM diethylamine NONOate (DEANO), a fast-release NO· donor, and the cellular manganese content was monitored over the course of 60 min. Consistent with prior observations, manganese levels increased by 30 min posttreatment and then returned to pretreatment levels by 60 min (Fig. 2) (14). Notably, manganese levels in the Δ*mntP* strain were not significantly different than in the wild-type at any time. These data indicate either that manganese efflux does not occur via MntP following NO· treatment or that, in the absence of *mntP*, manganese efflux occurs via another transporter in *S*. Typhimurium.

### YiiP expression enhances zinc toxicity in *S*. Typhimurium.

**FIG 3:**
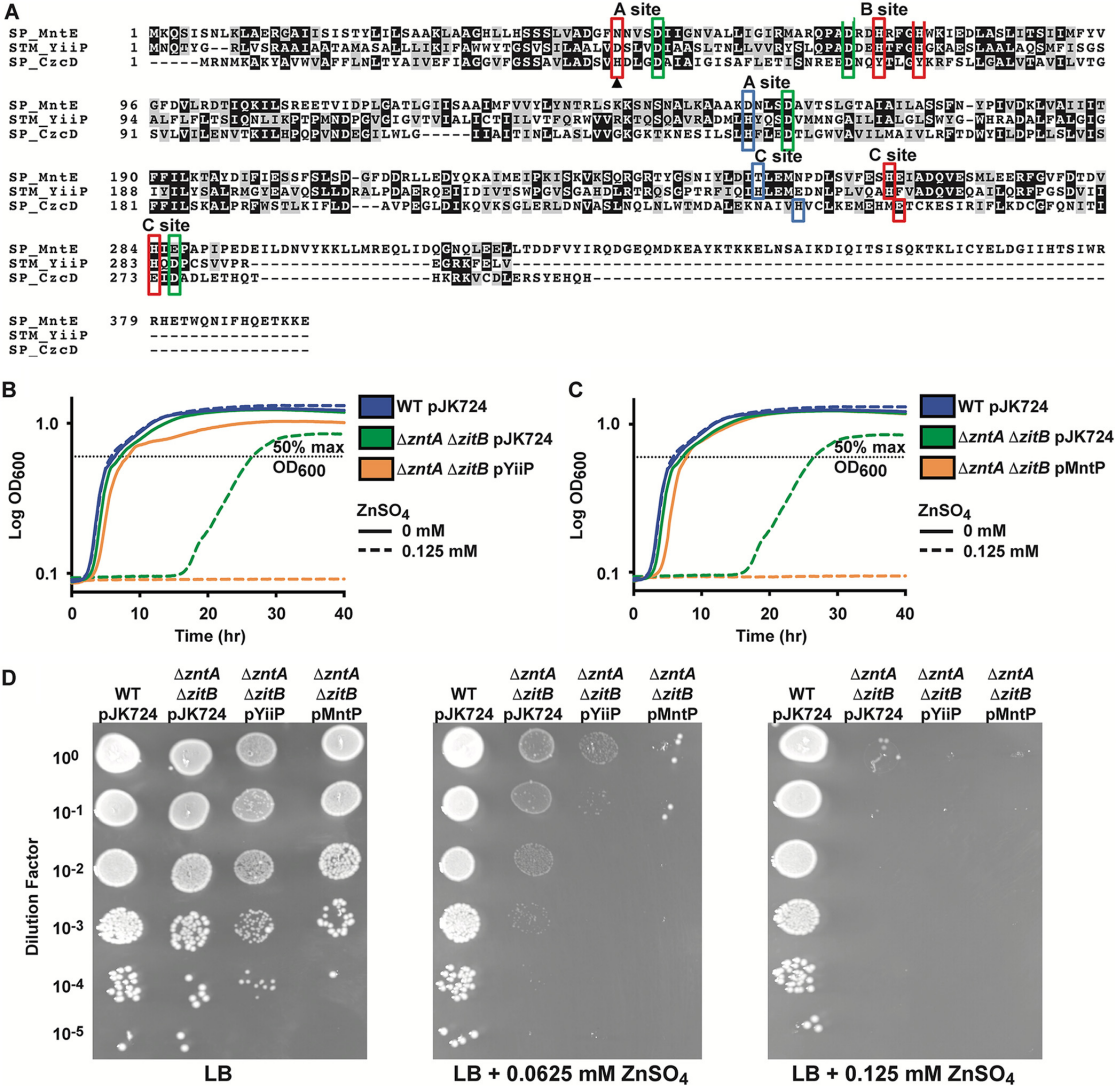
Expression of YiiP, a protein with homology to MntE and CzcD, enhances zinc sensitivity of an *S*. Typhimurium zinc efflux mutant strain. (A) Alignment of amino acid sequences for *S*. Typhimurium YiiP (STM_YiiP) and the related CDF proteins S. pneumoniae MntE (SP_MntE, SP_1552) and S. pneumoniae CzcD (SP_CzcD, SP_1857). Identical residues are shown in black, while similar residues are shown in gray. Metal-binding sites from the YiiP crystal structure (PDB ID: 2QFI) are shown. Ligands shared by all proteins are in green boxes. Red boxes denote ligands shared between YiiP and MntE. Blue boxes denote ligands shared between YiiP and CzcD. Boxes are staggered where the amino acid sequence alignment did not match previously assigned locations for two CzcD C-site residues. (B) Growth of *S*. Typhimurium wild-type (WT) pJK724, Δ*zntA* Δ*zitB* pJK724, and Δ*zntA* Δ*zitB* pYiiP strains in LB with or without 0.125 mM ZnSO_4_ supplementation. The Δ*zntA* Δ*zitB* pJK724 strain was delayed exiting lag phase compared to WT pJK724 in 0.125 mM ZnSO_4_ (*P* < 0.001), while the Δ*zntA* Δ*zitB* pYiiP strain failed to grow. (C) Growth of S. Typhimurium wild-type (WT) pJK724, Δ*zntA* Δ*zitB* pJK724, and Δ*zntA* Δ*zitB* pMntP strains in LB with or without 0.125 mM ZnSO_4_ supplementation. Data for the growth curves (B) and (C) are the mean of 3 independent experiments. Statistical significance of the difference between the mutant strains and the wild-type was determined using the time (hr) to reach 50% maximum growth (OD_600_; dashed line) and an unpaired two-tailed *t* test. (D) Growth of strains from (B) and (C) were assessed by spot assays. Dilutions of OD_600_ = 0.3 cultures were spotted onto LB agar supplemented with ZnSO_4_ as shown. Zinc efflux mutants expressing YiiP or MntP show decreased survival in the presence of ZnSO_4_. A representative spot assay for each condition is shown, selected from 3 independent replicates.

Since the *S*. Typhimurium genome contained only one MntP homologue, the genome was next searched for proteins with homology to the CDF family manganese transporter MntE. Two *S*. Typhimurium proteins were observed to have similarity to S. pneumoniae MntE. YiiP (STM4061) had the greatest similarity with 28% identity and 50% similarity over 264 amino acids. In addition to homology with MntE, YiiP also shares homology (26% identity and 51% similarity over 183 amino acids) with CzcD, a well-studied CDF family zinc transporter (Fig. 3A). ZitB (STM0758), a zinc transporter in E. coli and *S*. Typhimurium, had 20% identity and 48% similarity over 201 amino acids compared to S. pneumoniae MntE (28, 29).

E. coli YiiP, also known as FieF, was first reported to serve as an iron efflux transporter. Subsequent *in vitro* studies showed that YiiP also had the ability to interact with zinc ions, although its physiological contribution to zinc homeostasis remains to be defined (30–33). Three metal binding regions, the A-site, B-site, and C-site (comprised of C1 and C2 plus a linker) have been identified based on the E. coli YiiP crystal structure (31, 32, 34). The A-site has been established as the primary motif determining metal selectivity (34, 35). S. pneumoniae MntE has an ND-DD A-site motif, but DD-DD A-site motifs are also common in manganese exporting CDF proteins (36). By contrast, the zinc exporting CDF from S. pneumoniae, CzcD, has an HD-HD motif, which is the most common motif in CDF family zinc transporters (36). *S*. Typhimurium YiiP has a DD-HD A-site motif, which precludes bioinformatic prediction of the native ligand but is suggestive of the potential to export ions other than zinc.

Zinc sensitivity phenotypes have not been observed for *S*. Typhimurium or E. coli strains with *yiiP* deleted alone or in combination with other known zinc exporters (28, 29, 37). However, these studies could have failed to detect zinc sensitivity phenotypes if *yiiP* was not expressed under the conditions tested. To address this, we expressed *yiiP* constitutively from a low-copy-number plasmid (pYiiP) in the *S*. Typhimurium Δ*zntA* Δ*zitB* background to ascertain whether this could decrease the zinc sensitivity of this mutant strain. We observed that the Δ*zntA* Δ*zitB* strain was delayed for growth in the presence of 0.125 mM ZnSO_4_ compared to the wild-type. Expression of *yiiP* in the Δ*zntA* Δ*zitB* genetic background abrogated bacterial growth (Fig. 3B). Notably, expression of *mntP* elicited a similar phenotype in the Δ*zntA* Δ*zitB* background (Fig. 3C). Spot assays revealed that expression of either YiiP or MntP in the Δ*zntA* Δ*zitB* genetic background led to decreased survival in the presence of 0.0625 mM ZnSO_4_ and little to no survival on plates with 0.125 mM ZnSO_4_ (Fig. 3D). These data indicate that YiiP does not facilitate zinc export. It therefore follows that the increased sensitivity of the *yiiP*-expressing Δ*zntA* Δ*zitB* strain suggests that YiiP may export a different metal ion that results in enhanced susceptibility to zinc intoxication.

### Metal availability results in altered expression of *mntP* but not *yiiP*.

**FIG 4:**
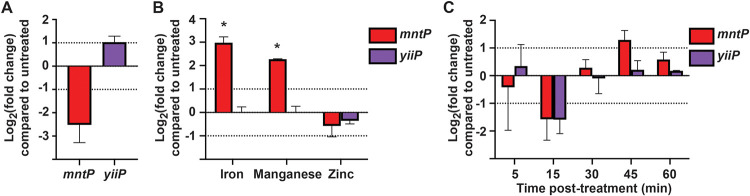
Expression of *mntP* increases in response to iron and manganese treatment, but *yiiP* does not. Log_2_ fold change in expression for treated *S*. Typhimurium wild-type cultures, by comparison with untreated. Dotted lines represent the thresholds for significant expression change. (A) Expression of *mntP* and *yiiP* in LB supplemented with 3 mM EDTA. The apparent decrease in *mntP* expression failed to achieve statistical significance. (B) Expression of *mntP* in Tris minimal medium increased in response to 0.5 mM iron (*P* = 0.014) and 0.5 mM manganese (*P* < 0.001). (C) Expression of *mntP* and *yiiP* in LB with 2 mM DEANO. No statistically significant expression changes occurred. Data are the mean of 3 (A and B) or 4 (C) independent experiments. Error bars represent standard deviation. Statistical significance (*) was determined by a one-sample *t* test to a hypothetical mean of either 1 or −1.

We next measured *yiiP* expression under conditions of metal limitation, metal stress, and NO· challenge compared to *mntP*. In the presence of general divalent cation chelator ethylenediaminetetraacetic acid (EDTA), expression of neither *mntP* nor *yiiP* was significantly altered (Fig. 4A). In response to metal stress, *mntP* was upregulated when the medium was supplemented with 0.5 mM iron or manganese, but not zinc (Fig. 4B). By contrast, *yiiP* expression did not change in response to iron, manganese, or zinc (Fig. 4B), which also differs from observations of the orthologous gene from E. coli (29, 30). Expression of neither *mntP* nor *yiiP* was significantly altered at any time following treatment with 2 mM DEANO (Fig. 4C).

### YiiP contributes to manganese resistance in *S*. Typhimurium.

**FIG 5:**
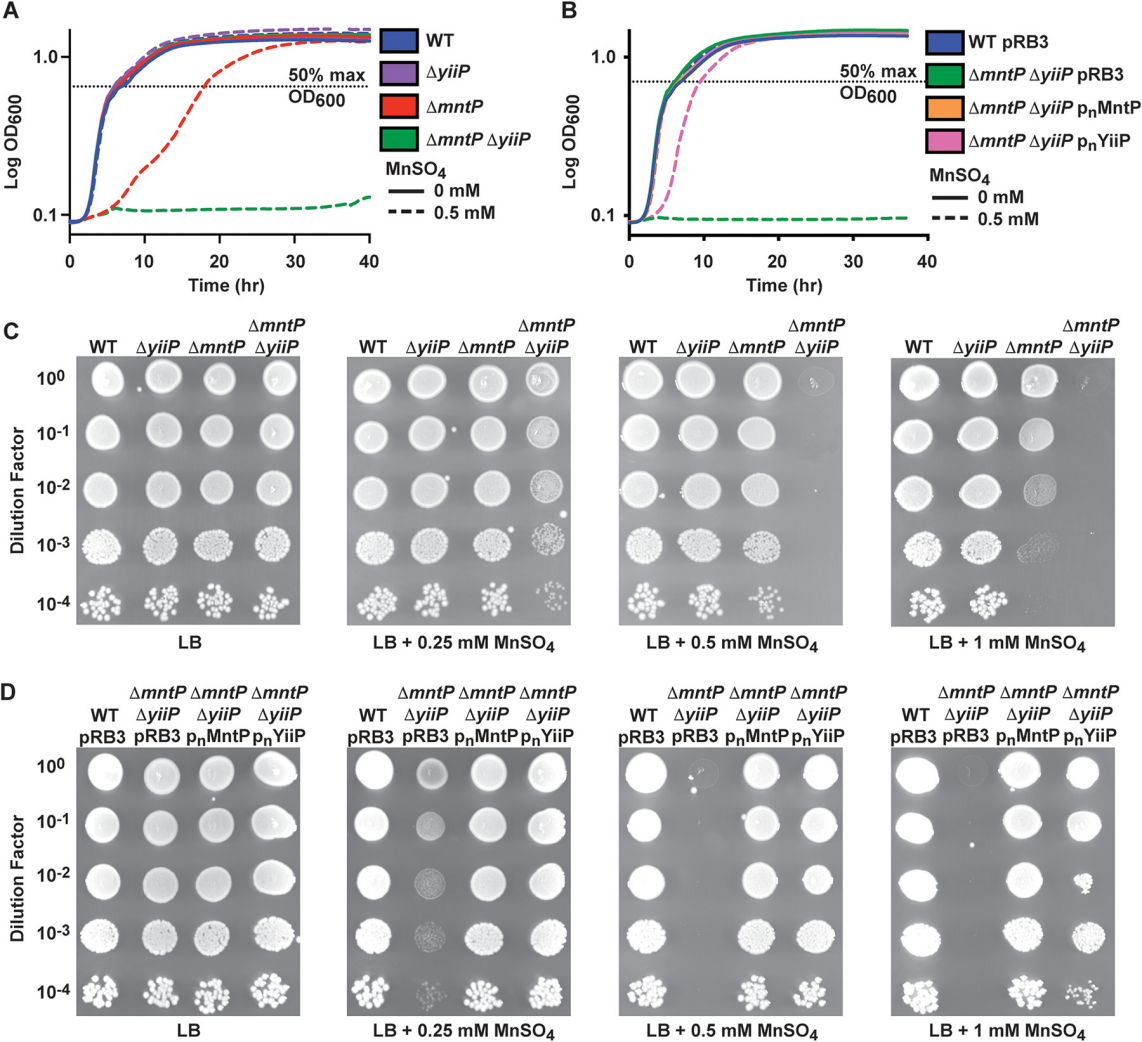
Manganese sensitivity of *S*. Typhimurium is enhanced in the absence of both *mntP* and *yiiP*. (A) Growth phenotypes of *S*. Typhimurium wild-type (WT), Δ*mntP*, Δ*yiiP*, and Δ*mntP* Δ*yiiP* strains in LB with or without 0.5 mM MnSO_4_ supplementation. The Δ*mntP* strain was delayed exiting lag phase compared to WT in LB supplemented with 0.5 mM MnSO_4_ (*P* < 0.001), and the Δ*mntP* Δ*yiiP* strain failed to grow. (B) Growth phenotypes of *S*. Typhimurium empty vector strains WT pRB3 and Δ*mntP* Δ*yiiP* pRB3 compared to plasmid-based complementation strains Δ*mntP* Δ*yiiP* p_n_MntP and *mntP* Δ*yiiP* p_n_YiiP in LB with or without 0.5 mM MnSO_4_ supplementation. The expression constructs p_n_MntP and p_n_YiiP use the native promoters. Growth of *mntP* Δ*yiiP* p_n_YiiP was delayed relative to WT (*P* < 0.001). Data for the growth curves (A) and (B) are the mean of 3 independent experiments. Statistical significance of the difference between the mutant strains and the wild-type was determined using the time (hr) to reach 50% maximum growth (OD_600_; dashed line) and an unpaired two-tailed *t* test. (C) Growth of strains from (A) by spot plate assays. Dilutions of OD_600_ = 0.3 cultures were spotted onto LB agar supplemented with MnSO_4_ as shown. The Δ*mntP* Δ*yiiP* strain displayed decreased growth at 0.25 mM MnSO_4_ and decreased survival at 0.5 mM and 1 mM. (D) Growth of strains from (B) by spot plate assays. Dilutions of OD_600_ = 0.3 cultures were spotted onto LB agar supplemented with MnSO_4_ as shown. A representative spot assay for each condition is shown, selected from 3 independent replicates. Expression of either YiiP or MntP complemented the survival defect of the Δ*mntP* Δ*yiiP* stain in the presence of MnSO_4_.

Although expression of *yiiP* did not change in response to metal stress under our experimental conditions, the protein may be present due to constitutive expression and contribute to metal ion efflux. Building on the observations that YiiP shares homology with MntE (Fig. 3A) and its expression enhanced the zinc sensitivity of a zinc efflux mutant strain in a similar fashion to expression of MntP (Fig. 3B to D), a role in manganese efflux was investigated. To assess whether YiiP contributes to manganese resistance of *S*. Typhimurium, Δ*yiiP* and Δ*mntP* Δ*yiiP* strains were generated. The Δ*yiiP* strain grew similarly to the wild-type in the presence of 0.5 mM MnSO_4_ (Fig. 5A). However, supplementation of the growth medium with 0.5 mM MnSO_4_ delayed the growth of the Δ*mntP* strain and abrogated growth of the Δ*mntP* Δ*yiiP* strain (Fig. 5A). In spot assays, the Δ*mntP* Δ*yiiP* strain displayed decreased growth at 0.25 mM MnSO_4_ and decreased survival at both 0.5 mM and 1 mM MnSO_4_. By contrast, the Δ*mntP* strain only showed a moderate decrease in survival at 1 mM MnSO_4_ (Fig. 5C). Taken together, these data show an enhancement of manganese sensitivity when both transporters are absent. Plasmid-based complementation with either *mntP* or *yiiP* expressed from the native promoter attenuated the growth defect of a Δ*mntP* Δ*yiiP* mutant, although the Δ*mntP* Δ*yiiP* p_n_YiiP strain still had a minor growth delay relative to the wild-type and Δ*mntP* Δ*yiiP* p_n_MntP strains (Fig. 5B). Similar complementation results were obtained in spot assays (Fig. 5D).

### *S*. Typhimurium mutants lacking *mntP* and *yiiP* are neither sensitive to zinc nor disrupted for zinc homeostasis.

Previous experiments investigating the metal binding properties of recombinant E. coli YiiP revealed a capacity to interact with zinc ions *in vitro*, but not manganese (33, 38). Thus, the observed impact of manganese on the growth and survival of the *S*. Typhimurium Δ*mntP* Δ*yiiP* strain may reflect an indirect effect on zinc homeostasis. Interplay between zinc and manganese homeostasis has been shown to occur in several Gram-positive pathogens, such as S. pneumoniae where zinc has been established to disrupt manganese uptake and increase sensitivity to oxidative stress (39–42). However, this phenomenon has not been reported for the *Enterobacteriaceae* and, in *S*. Typhimurium, this may be attributable to the presence of the manganese-transporting natural resistance-associated macrophage protein (NRAMP) transporter MntH, which is not susceptible to zinc inhibition (9). By contrast, the impact of manganese on zinc homeostasis in *S*. Typhimurium has not been determined. Accordingly, we investigated whether the observed manganese sensitivity of the *S*. Typhimurium Δ*mntP* Δ*yiiP* strain was due to pleiotropic effects of perturbed zinc homeostasis.

**FIG 6:**
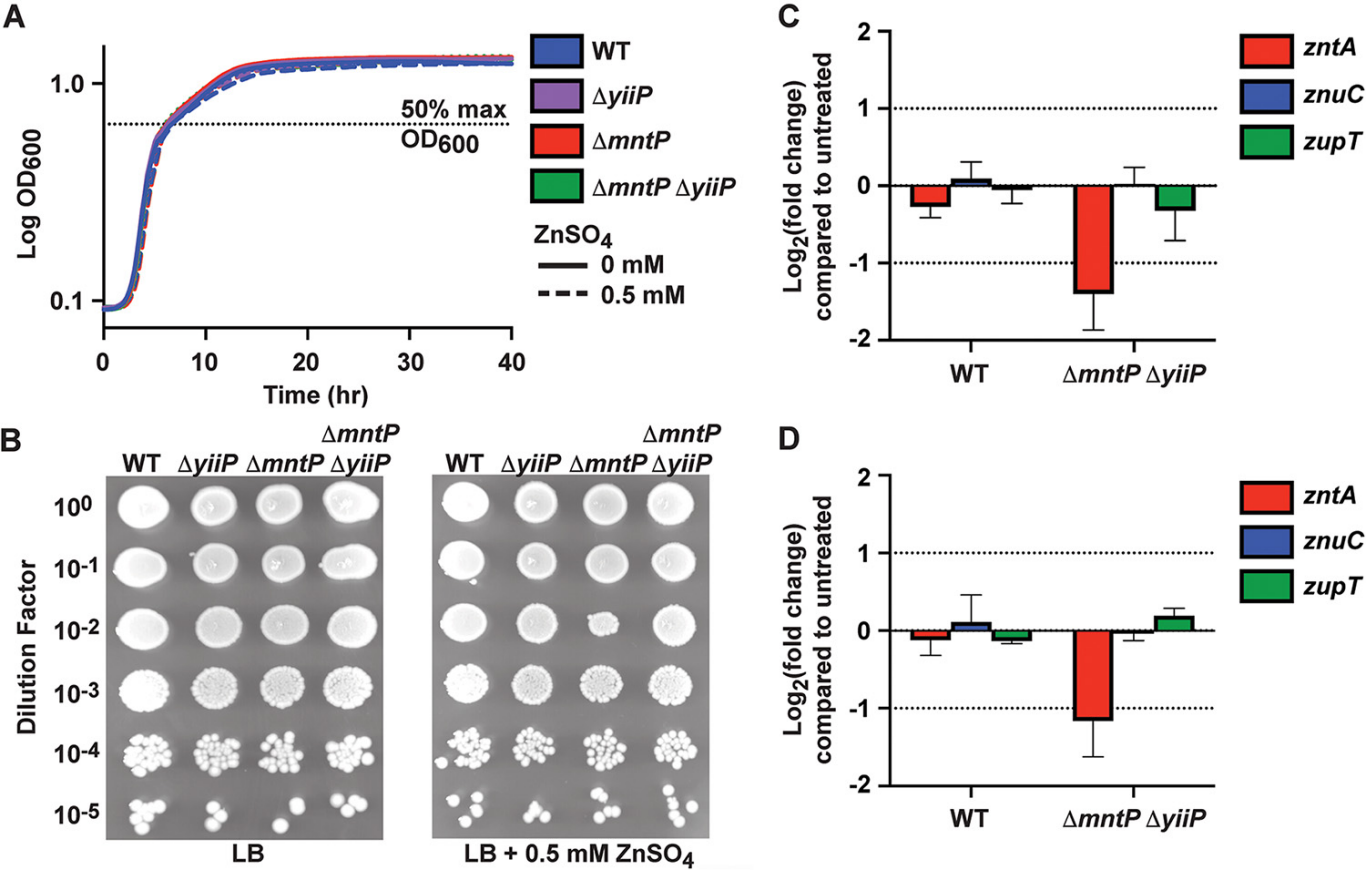
Zinc sensitivity is not enhanced in a Δ*mntP* Δ*yiiP* mutant, and expression of zinc-regulated transporters is not perturbed by manganese. (A) Growth of *S*. Typhimurium wild-type (WT) and the mutant derivatives Δ*yiiP*, Δ*mntP*, and Δ*mntP* Δ*yiiP* in LB with or without 0.5 mM ZnSO_4_ supplementation. Data are the mean of 3 independent experiments, and statistical significance was determined by the time (hr) to reach 50% maximum growth (OD_600_; dashed line) by unpaired two-tailed *t* test. (B) Growth of strains from (A) assessed using spot assays on LB agar supplemented with ZnSO_4_ as shown. A representative spot assay for each condition is shown, selected from 3 independent replicates. (C) qPCR analyses of the zinc-regulated genes *zntA*, *znuC*, and *zupT* determined in response to challenge by 0.5 mM MnSO_4_ (20 min exposure; OD_600_ = 0.5 cultures). (D) qPCR analyses of the zinc-regulated genes *zntA*, *znuC*, and *zupT* determined in response to challenge by 0.5 mM MnSO_4_ (60 min exposure; OD_600_ = 0.5 cultures). Data in (C) and (D) are the mean (± standard deviation) of 3 independent experiments. Statistical significance was determined by a one-sample *t* test to a hypothetical mean of either 1 or −1. No significant differences were observed.

Here, we examined the impact of zinc stress on the wild-type Δ*yiiP*, Δ*mntP*, and Δ*mntP* Δ*yiiP* strains. This revealed that zinc supplementation had no impact on the growth phenotype of any strain (Fig. 6A and B). These data indicate that *S*. Typhimurium zinc homeostasis is not dysregulated in the absence of manganese efflux. Furthermore, neither YiiP nor MntP contributes to substantially to zinc homeostasis. To further probe the impact on the zinc regulatory network of *S*. Typhimurium, the expression of genes controlled by the zinc uptake regulator, Zur, and zinc export regulator, ZntR, were analyzed in the wild-type and Δ*mntP* Δ*yiiP* strains during exposure to excess manganese. The sensitivity of these metalloregulators is in the femtomolar to nanomolar range; thus, they provide a highly sensitive insight into cellular zinc homeostasis (43–45). Here, we monitored the expression of the Zur-regulated zinc uptake transporter *znuABC*, the primary *S*. Typhimurium pathway for zinc acquisition (46–48); the ZntR-regulated zinc exporter *zntA*, the major *S*. Typhimurium zinc efflux system (28, 49, 50); and *zupT*, which has been implicated in zinc and manganese import, although its regulatory control remains to be defined (14, 51, 52). These data show that there was no significant difference in expression of *zntA*, *znuC*, or *zupT* between the wild-type and Δ*mntP* Δ*yiiP* strains at 20 min (Fig. 6C) or 60 min (Fig. 6D) in the presence of manganese. Thus, the *S*. Typhimurium Δ*mntP* Δ*yiiP* manganese-induced growth and survival defects (Fig. 5) are not an indirect effect arising from disrupted zinc homeostasis.

### YiiP contributes to manganese efflux in *S*. Typhimurium.

**FIG 7:**
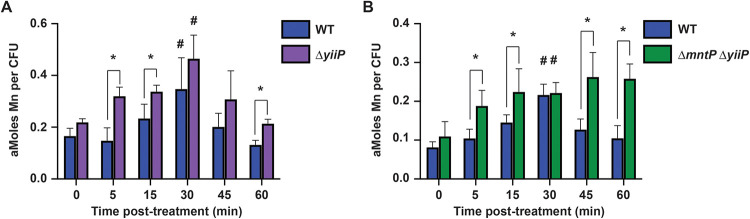
Intracellular manganese levels increase sooner in Δ*yiiP* and Δ*mntP* Δ*yiiP* mutants following NO· treatment and do not decrease in Δ*mntP* Δ*yiiP*. Total cellular manganese accumulation in wild-type (WT) and mutant *S*. Typhimurium cultures treated with 2 mM DEANO, determined by ICP-MS. (A) Intracellular manganese was greater in Δ*yiiP* *S*. Typhimurium than in WT (*) at 5 (*P* = 0.004), 15 (*P* = 0.03) and 60 min (*P* = 0.001) posttreatment. Manganese was elevated (#) in both WT (*P* = 0.02) and Δ*yiiP* (*P* = 0.009) at 30 min posttreatment compared to 0 min. (B) Intracellular manganese was greater in Δ*mntP* Δ*yiiP* *S*. Typhimurium than in WT (*) at 5 (*P* = 0.01), 15 (*P* = 0.04), 45 (*P* = 0.008), and 60 min (*P* < 0.001) posttreatment. Manganese was significantly elevated (#) in WT (*P* < 0.001) and Δ*mntP* Δ*yiiP* (*P* = 0.003) at 30 min posttreatment compared to 0 min. In WT, intracellular manganese was lower at 45 min than at 30 min (*P* = 0.003) and was no different than at 0 min by 60 min posttreatment. Intracellular manganese was no different in Δ*mntP* Δ*yiiP* at 45 and 60 min posttreatment than at 30 min. ICP-MS data are the mean of 4 independent experiments. Error bars represent standard deviation. Statistically significant differences across time points for each strain and between WT and mutant were determined by unpaired two-tailed *t* test.

We next investigated whether YiiP participates in control of cellular manganese levels following exposure to NO·. It is important to note that in this experiment, direct comparisons are confined to the wild-type and the mutant strains within each analysis. This is due to differences in medium manganese concentrations that differed between the analyses (i.e., Fig. 7A vs. Fig. 7B), which influenced the absolute cellular abundances in the bacterial strains (43). Changes in cellular manganese followed generally similar patterns in the wild-type and Δ*yiiP* strains following treatment with 2 mM DEANO. However, manganese levels were elevated in the Δ*yiiP* strain relative to the wild-type strain at 5, 15, and 60 min (Fig. 7A). Manganese levels were greater in the Δ*mntP* Δ*yiiP* strain than in the wild-type at 5, 15, 45, and 60 min after treatment with 2 mM DEANO, but both strains reached a similar peak at 30 min (Fig. 7B). In the wild-type strain, cellular manganese decreased at 45 min compared to the 30 min peak and fell to pretreatment levels by 60 min. In the Δ*mntP* Δ*yiiP* strain, there was no significant difference in manganese levels at 30, 45, and 60 min posttreatment, suggesting that efflux was impaired when both genes were deleted (Fig. 7B).

### Residue Asp45 is required for YiiP to alleviate manganese toxicity.

**FIG 8:**
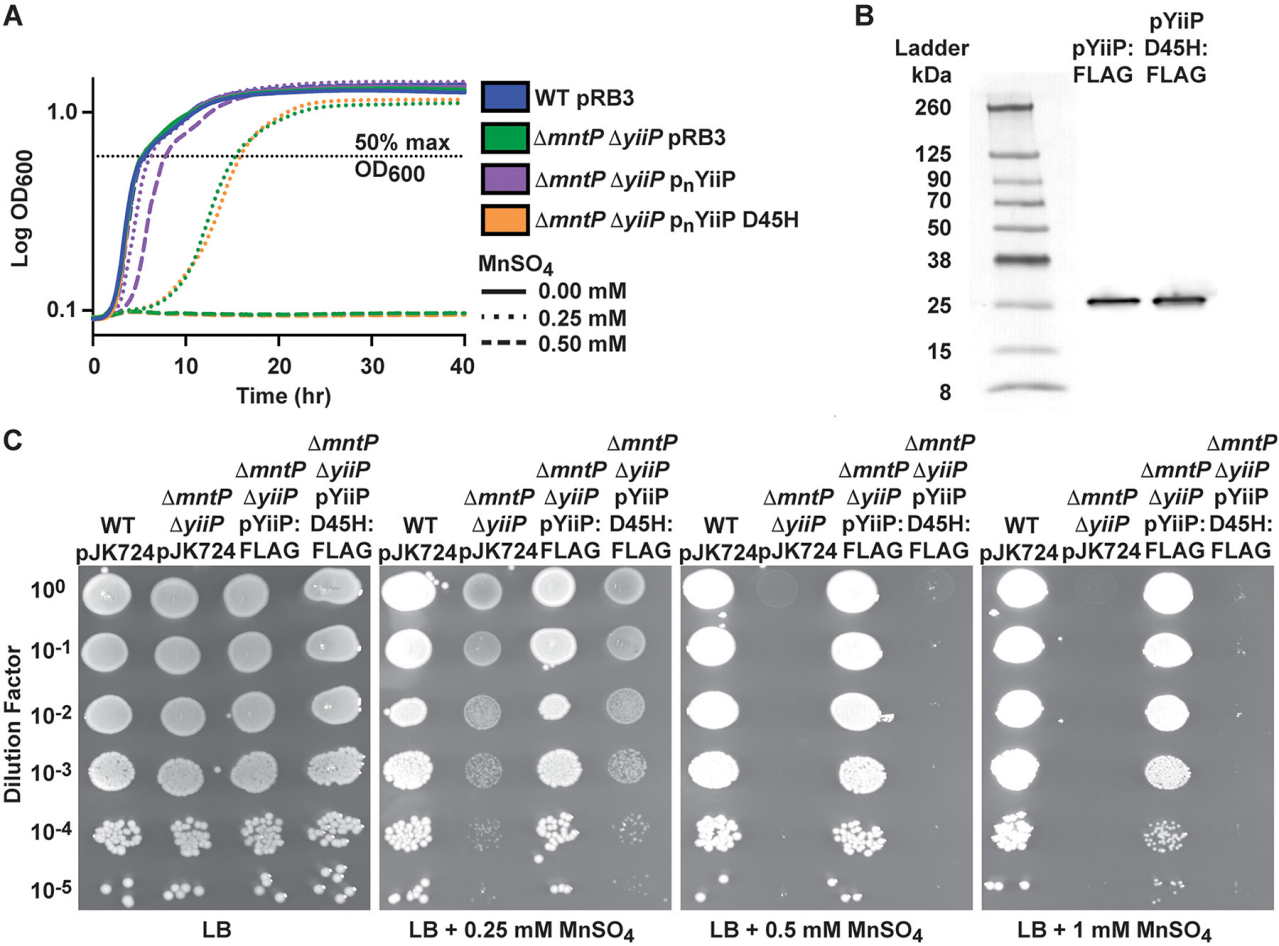
An aspartate residue at position 45 is required for manganese protection by YiiP. (A) Growth phenotypes of *S*. Typhimurium wild-type (WT pRB3), Δ*mntP* Δ*yiiP* pRB3, Δ*mntP* Δ*yiiP* p_n_YiiP, and Δ*mntP* Δ*yiiP* p_n_YiiP D45H strains in LB with or without MnSO_4_ supplementation. The Δ*mntP* Δ*yiiP* pRB3 and Δ*mntP* Δ*yiiP* p_n_YiiP D45H strains failed to grow in 0.5 mM MnSO_4_. In 0.25 mM MnSO_4_ there was no difference between Δ*mntP* Δ*yiiP* pRB3 and Δ*mntP* Δ*yiiP* p_n_YiiP D45H. Both strains were delayed for growth in 0.25 mM MnSO_4_ compared to WT pRB3 (*P* < 0.001) and Δ*mntP* Δ*yiiP* p_n_YiiP (*P* < 0.001). Data are the mean of 3 independent experiments, and statistical significance was determined by the time (hr) to reach 50% maximum growth (OD_600_; dashed line) by unpaired two-tailed *t* test. (B) Western blot of FLAG-tagged YiiP and YiiP D45H expressed in *S*. Typhimurium Δ*mntP* Δ*yiiP* shown adjacent to a visible protein standard. Single bands of equal intensity were observed for both versions of the YiiP protein at an apparent size of ∼26 kDa. (C) Spot plate assays of *S*. Typhimurium WT, Δ*mntP* Δ*yiiP*, and the Δ*mntP* Δ*yiiP* complemented strains encoding FLAG-tagged fusion variants of YiiP and YiiP D45H on LB with and without MnSO_4_ supplementation. A representative spot assay for each condition is shown, selected from 3 independent replicates. The Δ*mntP* Δ*yiiP* strain expressing YiiP D45H:FLAG displays the same phenotype as the Δ*mntP* Δ*yiiP* empty vector strain.

Prior work has indicated that the A-site motif of CDF transporters dictates metal specificity (34, 35). For YiiP, the aspartate residue at position 45 has been implicated in facilitating transport of both cadmium and zinc in *in vitro* assays (35). When the aspartate residue was mutated to histidine (D45H), creating an HD-HD A-site motif in YiiP, the mutant protein transported zinc at similar rates as wild-type YiiP in *in vitro* assays, but no longer transported cadmium (35). In this study, our data indicate that *S*. Typhimurium YiiP is associated with physiological manganese export and do not support a role in zinc homeostasis; however, this does not preclude a capacity for interaction with zinc *in vitro*. Here, we sought to determine the contribution of Asp45 to YiiP manganese transport. We generated a point mutation, substituting histidine for aspartate (D45H) in the p_n_YiiP plasmid (p_n_YiiP D45H). The mutant derivative was then investigated in a Δ*mntP* Δ*yiiP* background grown in medium supplemented with 0.5 mM MnSO_4_. We observed that the growth phenotype of the Δ*mntP* Δ*yiiP* p_n_YiiP D45H strain was no different than the Δ*mntP* Δ*yiiP* pRB3 strain (empty vector) in the presence of manganese, indicating a lack of complementation (Fig. 8A). To confirm that lack of complementation was due to lack of transporter function rather than protein instability, FLAG-tagged YiiP and YiiP D45H were expressed constitutively in the Δ*mntP* Δ*yiiP* background, subjected to SDS-PAGE and visualized by Western blot. Single bands of similar intensity were observed for both proteins at ∼26 kDa (Fig. 8B). Although this is smaller than the predicted mass for YiiP (32.9 kDa), integral membrane proteins are known to show altered mass profiles in SDS-PAGE analyses. To confirm that the FLAG-tagged YiiP proteins were functional, spot assays were performed. Expression of YiiP:FLAG complemented the growth and survival defects of the Δ*mntP* Δ*yiiP* strain while expression of pYiiP D45H:FLAG did not (Fig. 8C). Collectively, these results show that Asp45 is required for manganese transport activity by *S*. Typhimurium YiiP.

## DISCUSSION

Manganese efflux by MntP and the CDF transporter MntE has been studied in a variety of prokaryotic species, but thus far the presence of multiple manganese efflux systems within a single prokaryote has only been characterized in B. subtilis. *S*. Typhimurium encodes both an MntP homologue and YiiP, a CDF transporter. Identifying the target metal of CDF family transporters can be challenging despite efforts to establish the motifs that provide ion selectivity. YiiP has homology within the metal coordinating A-site to both zinc and manganese-transporting members of the CDF family (Fig. 3A) (36). YiiP from E. coli has been characterized as a zinc transporter using *in vitro* and structural methods, while an *in vivo* study linked its function to iron homeostasis. A physiological role for YiiP in zinc homeostasis has not yet been established in *S*. Typhimurium or E. coli, with Δ*yiiP* mutant strains not demonstrating zinc susceptible phenotypes (28, 29, 37). Here we show that expression of YiiP enhances the zinc sensitivity of Δ*zntA* Δ*zitB* *S*. Typhimurium rather than reducing it (Fig. 3B). These data, along with those showing enhanced manganese sensitivity and lack of manganese export in Δ*mntP* Δ*yiiP* *S*. Typhimurium (Fig. 5, 7B), suggest that zinc is not the cognate cargo of *S*. Typhimurium YiiP *in vivo*. Manganese transport phenotypes have been demonstrated *in vivo* for YiiP homologues from Sinorhizobium meliloti, Deinococcus radiodurans, and Rhizobium etli (53–55). These proteins share a clade with YiiP from E. coli that is phylogenetically distinct from the clades containing the well-characterized CDF zinc transporters CzcD and ZitB (54, 56). The variety of proposed substrates for transporters within this clade is diverse while relatively few transporters have been experimentally characterized. Further work will be required to gain an accurate understanding of the biological functions of this group of transporters.

While function of MntP alone was sufficient to provide protection from manganese toxicity under the conditions of this study, deletion of both *mntP* and *yiiP* enhanced the sensitivity of *S*. Typhimurium to manganese intoxication (Fig. 5A and C). Function of either MntP or YiiP could restore manganese homeostasis following NO· treatment, and only the Δ*mntP* Δ*yiiP* strain was abrogated for manganese efflux during the late-stage response to NO· exposure (Fig. 7B). Selectivity for manganese depends on residue Asp45 within the A-site metal binding region of YiiP. A change in transport activity in response to mutation of Asp45 is consistent with similar results arising from the mutation of A-sites in E. coli YiiP and other CDF transporters (34, 35). While we cannot exclude the possibility that YiiP might facilitate the transport of other transition metal cations under certain conditions, the results of this study support a role for YiiP as a manganese exporter in *S*. Typhimurium.

Regulation of *yiiP* expression remains an open question. Unlike *mntP*, it is not part of the MntR regulon and has not been shown to be part of the Fur, Zur, or ZntR regulons (26, 57). Expression of *mntP* is regulated by both MntR and the *ybbP-ykoY* manganese-sensing riboswitch in E. coli (58). The promoter region of *mntP* from *S*. Typhimurium shows homology to that of E. coli, suggesting that similar regulatory mechanisms may apply to *mntP* expression in both organisms. By contrast, the promoter region of *yiiP* lacks this homology. Induction of *mntP* expression occurred in response to manganese and iron stress, whereas *yiiP* expression was unaffected by the conditions tested (Fig. 4B). Previous work using β-galactosidase fusions to the *yiiP* promoter in E. coli showed upregulation in response to zinc and a modest response to iron after several hours of incubation (29, 30). Since none of the known metal-responsive regulators have been implicated in *yiiP* expression, any expression change in response to metal excess may be indirect and more time may be necessary for the cellular conditions driving regulation to develop. Alternatively, expression of *yiiP* may be constitutive in *S*. Typhimurium under standard laboratory growth conditions. Constitutive expression would be consistent with the more rapid accumulation of manganese observed in strains lacking *yiiP* (Fig. 7), while a strain lacking only the inducible *mntP* acquires manganese similarly to the wild-type (Fig. 2). Expression of *mntE*, the CDF manganese transporter from S. pneumoniae, does not respond to manganese abundance and has been proposed to be expressed constitutively (19). Manganese efflux activity in E. coli is further regulated by the small protein MntS. E. coli that overexpress MntS display enhanced manganese accumulation and have the same manganese-sensitive phenotype as Δ*mntP* mutants, suggesting that this small protein may function to prevent the efflux activity of MntP (16). Overexpression of MntS in a Δ*mntP* strain did not enhance the manganese sensitivity of this mutant, suggesting that it does not inhibit the function of additional manganese exporters such as YiiP, but this possibility has not yet been investigated directly.

While efflux by either MntP or YiiP is sufficient to restore baseline manganese levels in *S*. Typhimurium following NO· exposure, and both contribute to protecting *S*. Typhimurium from excess manganese in culture, the broader functional significance of these transporters in *S*. Typhimurium biology and pathogenesis has yet to be determined. Manganese acquisition is required for S. Typhimurium infection and virulence, but what role, if any, efflux might play is not currently known (10, 11). Manganese efflux has been shown to play an important role in the virulence of other pathogens such as S. pneumoniae and S. aureus and in colonization by E. faecalis (19–21). Although manganese has primarily been understood to act as an antioxidant, and absence of *mntE* rendered S. pneumoniae more resistant to both NO· and oxidative stress in the form of methyl viologen, this has not been the case for all organisms and oxidants (19). S. aureus lacking *mntE* was more sensitive to sodium hypochlorite (NaOCl), while S. pyogenes was more sensitive to peroxide (H_2_O_2_) (20, 22). Manganese excess has been shown to affect electron transport chain function and function of Fe-S cluster enzymes in central energy-generating pathways. (15–17). These proteins are also targets of oxidants, which could explain, at least in part, why enhanced sensitivity to oxidants is seen for some species, but the mechanisms underlying the requirement for manganese efflux remain incompletely understood. In light of these data, the dynamic flux of multiple metals that occurs in response to NO·, and the varied environments and stresses encountered by pathogens within the host, characterizing the biological roles of microbial manganese efflux systems, is an open and potentially complex area of investigation. Based on the findings of this study, we suggest that any future work concerning the biological significance of manganese efflux in *S*. Typhimurium should address both the function of MntP and the newly established manganese export function of YiiP.

## MATERIALS AND METHODS

### Bioinformatic analysis.

*S*. Typhimurium homology matches to MntP from Escherichia coli MG1655 and MntE and CzcD from Streptococcus pneumoniae TIGR4 were determined using BLASTP from the Kyoto Encyclopedia of Genes and Genomes (KEGG) website with default settings. Alignments were generated using the KEGG ClustalW tool (59). Alignment graphics were generated using BoxShade hosted by ExPASy, the Swiss Institute of Bioinformatics Resource Portal (https://embnet.vital-it.ch/software/BOX_form.html).

### Growth conditions.

Salmonella enterica serovar Typhimurium and Escherichia coli were grown in Luria-Bertani medium (LB; Fisher) at 37°C with shaking at 250 rpm unless otherwise specified. Antibiotic selection was used for strain construction only at the following concentrations: 100 μg mL^−1^ ampicillin (Amp), 50 μg mL^−1^ kanamycin (Kan), and 20 μg mL^−1^ chloramphenicol (Cm).

### Strain and plasmid construction.

All strains and plasmids are listed in Table 1. All primers are listed in Table 2. *S*. Typhimurium strains were generated in the ATCC 14028s genetic background, which also served as the wild-type strain in this study (JK237). Deletion mutants were constructed using λ-RED mediated recombination with either pKD3 or pKD4 as the template (60). Expression of Flp-FRT recombinase from pCP20 was used to remove the antibiotic resistance cassettes from strains EF657 and EF635 to generate strains EF725 and EF755 (60). Combination mutants were created using P22HT105/int bacteriophage transduction (61). All mutants were verified by PCR.

**TABLE 1:** Strains and plasmids

Plasmid or strain	Genotype	Source
pKD46	*bla araC-*P_araB_-γβ *exo* oriR101 repA101ts	(60)
pKD3	*bla FRTcatFRT* PS1 PS2 oriRγ	(60)
pKD4	*bla FRTaphFRT* PS1 PS2 oriRγ	(60)
pCP20	*bla cat* cI857 IPr *flp* PSC101 oriTS	(60)
pRB3-273C (pRB3)	*bla par* RK2 oriV *trfA*	(62)
pJK724	*bla par* RK2 oriV *tfrA* P_trc_	(63)
pEF101 (p_n_MntP)	bla par RK2 oriV tfrA P_native_-mntP	This study
pEF102 (p_n_YiiP)	bla par RK2 oriV tfrA P_native_-yiiP	This study
pEF103 (pYiiP)	bla par RK2 oriV tfrA P_trc_-yiiP	This study
pEF106 (pMntP)	*bla par* RK2 oriV *tfrA* P_trc_-mntP	This study
pEF108 (p_n_YiiP D45H)	bla par RK2 oriV tfrA P_native_-yiiP D45H	This study
pEF115	bla par RK2 oriV tfrA P_trc_-yiiP:FLAG	This study
(pYiiP:FLAG)		
pEF116	bla par RK2 oriV tfrA P_trc_-yiiP D45H:FLAG	This study
(pYiiP D45H:FLAG)		
JK237 (WT)	14028s	ATCC
JK895(FLS187)	14028s/pRB3-273C	(65)
EF560	Δ*yiiP::frt-kan-frt*	This study
EF561	Δ*yiiP::frt-cm-frt*	(28)
EF564	14028s/pJK724	(14)
EF610	Δ*fljBA::FRT fliC5569::tetRA* (+1UTR)	(14)
EF635	Δ*mntP::frt-cm-frt*	This study
EF657	Δ*mntP::frt-cm-frt* Δ*yiiP::frt-kan-frt*	This study
EF697	Δ*fljBA::FRT fliC5569::tetRA* (+1UTR) Δ*mntP::frt-cm-frt*	This study
EF698	Δ*fljBA::FRT fliC5569::tetRA* (+1UTR) Δ*yiiP::frt-kan-frt*	This study
EF701	Δ*fljBA::FRT fliC5569::tetRA* (+1UTR) Δ*mntP::frt-cm-frt* Δ*yiiP::frt-kan-frt*	This study
EF725	Δ*mntP::FRT* Δ*yiiP::FRT*	This study
EF737	Δ*mntP::frt-cm-frt*/pRB3-273C	This study
EF738	Δ*mntP::frt-cm-frt*/pEF101	This study
EF739	Δ*mntP::frt-cm-frt* Δ*yiiP::frt-kan-frt*/pRB3-273C	This study
EF740	Δ*mntP::frt-cm-frt* Δ*yiiP::frt-kan-frt*/pEF101	This study
EF741	Δ*mntP::frt-cm-frt* Δ*yiiP::frt-kan-frt*/pEF102	This study
EF745	Δ*zntA::frt-cm-frt* Δ*zitB::frt-kan-frt/* pJK724	This study
EF746	Δ*zntA::frt-cm-frt* Δ*zitB::frt-kan-frt/* pEF103	This study
EF747	Δ*mntP::frt-cm-frt* Δ*yiiP::frt-kan-frt*/pJK724	This study
EF754	Δ*yiiP::frt-kan-frt*	This study
EF755	Δ*mntP::FRT*	This study
EF762	Δ*zntA::frt-cm-frt* Δ*zitB::frt-kan-frt/* pEF106	This study
EF773	Δ*mntP::frt-cm-frt* Δ*yiiP::frt-kan-frt*/pEF108	This study
EF829	Δ*mntP::frt-cm-frt* Δ*yiiP::frt-kan-frt*/pEF115	This study
EF830	Δ*mntP::frt-cm-frt* Δ*yiiP::frt-kan-frt*/pEF116	This study

**TABLE 2:** Primer sequences

Primer	Sequence 5′–3′	Purpose
EFP21	CGACATCATAACGGTTCTGGC	Sequencing of
		pEF103, pEF106
EFP300	ATGTTTGCTGGGGGCAGTGATGTGTTTAATGGATACCC CGGTGTAGGCTGGAGCTGCTTC	Creation of EF635
EFP301	TTAACCGTGAAAATGCGTCCAGAGGATCTGGACGCCAA	Creation of EF635
	TTCATATGAATATCCTCCTTAG	
EFP306	ATGTTAATGTTGCGCCGTCAATTGG	EF635 validation
EFP319	CACCCATGGCCATGAATCAAACCTATGGACGGC	Creation of pEF103
EFP320	CACAAGCTTTTATACAAGCTCGAACTTCCTGCC	Creation of pEF103
EFP334	GCGGGTACCAACTTCTTATTGAAAATCAATATC	Creation of pEF101
EFP335	ATAAAGCTTTTAACCGTGAAAATGCGTCCAG	Creation of pEF101, pEF106
EFP336	ATAGGTACCCCCTGTTTTCCTTGCCATAGACACC	Creation of pEF102
EFP340	GCGAAGCTTTTATACAAGCTCGAACTTCCTGCC	Creation of pEF102
EFP346	AAACCATGGCAATGTTTGCTGGGGGCAGTG	Creation of pEF106
EFP347	AATATCCACCAGCGAGTGCACCAACGC	Creation of pEF108
EFP397	CACAAGCTTTTACTTGTCGTCATCGTCTTTGTAGTCTAGCT	Creation of pEF115,
	CAACGAACTTCCTGCC	pEF116
JKP227	ACTCATTAGGCACCCCAGGC	Sequencing of
		pEF101, pEF102
JKP244	CTCTTCGCTATTACGCCAGC	Sequencing of
		pEF101, pEF102
JKP744	CCTATGGACGGCTGGTTAGCCGGGCGGCTATCGCGGCAACGTGTAGGCTGGAGCTGCTTC	Creation of EF560, EF561
JKP745	AATGACATCTGAACCCGGAAAACGCTGTAAAATCGCCTGCCATATGAATATCTCCTTAG	Creation of EF560, EF561
JKP746	TGAAAAGCATCAGCAACGAA	EF560, EF561 validation
JKP747	CCGATTTTCTTAATCATGACTACC	EF560, EF561 validation
JKP777	CACTTCTGAGTTCGGCATGG	Sequencing of
		pEF103, pEF106
rpoD qPCR fwd	GTGAAATGGGCACTGTTGAAC	*rpoD* qPCR
rpoD qPCR rev	TTCCAGCAGATAGGTAATGGC	*rpoD* qPCR
yiiP qPCR fwd	ACTCGCTGGTGGATATTGCC	*yiiP* qPCR
yiiP qPCR rev	ACAGAAACAACGCCGAACCG	*yiiP* qPCR
mntP qPCR fwd	GCGTACCGGTCTTATCTTTGG	*mntP* qPCR
mntP qPCR rev	GCTATCCAGTGGTTCCATTCC	*mntP* qPCR
zntA qPCR fwd	TCTGTATCCTATTGCCCGCC	*zntA* qPCR
zntA qPCR rev	CAATAAACAGCGCGCCAATG	*zntA* qPCR
znuC qPCR fwd	GGGGAAGTCAACGCTTGTAC	*znuC* qPCR
znuC qPCR rev	TTTGCGGGACATAGCCGATA	*znuC* qPCR
zupT qPCR fwd	GATCATGCTGCTTATCTCGCTG	*zupT* qPCR
zupT qPCR rev	CCAGATCCTGCGGATGAGCGTG	*zupT* qPCR

E. coli strain TB1 was used as the cloning host strain. Purified genomic DNA from *S*. Typhimurium 14028s was used as the PCR template unless otherwise specified. Plasmids pEF101 and pEF102 were generated by amplifying the upstream promoter region and coding sequence of *mntP* using primers EFP334 and EFP335 and *yiiP* using primers EFP336 and EFP340. Amplified fragments were digested with KpnI and HindIII then ligated into pRB3-273C digested with the same enzymes in reverse orientation to the multiple cloning site promoter (62). Constructs were confirmed by sequencing with JKP227 and JKP244 then transformed into strain EF657 to generate strains EF744 and EF745. To generate plasmids pEF103 and pEF106, the coding sequence of *yiiP* was amplified using primers EFP319 and EFP320 and the coding sequence of *mntP* was amplified using primers EFP346 and EFP335. Amplified fragments were digested with NcoI and HindIII then ligated into pJK724 digested with the same enzymes (63). Constructs were sequenced using primers EFP21 and JKP777 then transformed into zinc efflux mutant strain EF528 to generate strains EF746 and EF762. To generate plasmid pEF108, primers EFP336 and EFP347 were used to amplify a short fragment containing the target mutation (D45H). The fragment was purified from an agarose gel then used in a second amplification reaction with EFP340. Full-length product was digested with KpnI and HindIII then ligated into pRB3-273C digested with the same enzymes in reverse orientation to the multiple cloning site promoter. The construct was verified by sequencing with JKP227 and JKP244 then transformed into strain EF657 to generate strain EF773. Plasmids pEF115 and pEF116 were generated by amplifying the coding sequences of plasmids pEF102 and pEF108 using primers EFP319 and EFP397. Amplified fragments were digested with NcoI and HindIII then ligated into pJK724 digested with the same enzymes. Constructs were sequenced using primers EFP21 and JKP777 then transformed into strain EF657 to generate strains EF829 and EF830.

### Metal sensitivity growth curve assays.

To determine manganese sensitivity, wild-type *S*. Typhimurium (JK237) and transporter mutants (EF561, EF635, EF657) were grown overnight in LB, normalized to optical density at 600 nm (OD_600_) of 1, and diluted in triplicate 1:1000 into LB or LB with 0.5 mM MnSO_4_ for a final volume of 300 μL in a flat-bottom 96-well nontreated tissue culture microtiter plate (Midwest Scientific). Cultures were grown aerobically with shaking at 567 rpm (3 mm) in a Biotek Synergy HTX multimode 96-well plate reader at 37°C. Growth was monitored by recording OD_600_ every 15 min for 40 h. Statistical significance was determined by comparing the time required to reach 50% maximum OD_600_ by unpaired two-tailed *t* test using Microsoft Excel.

Complementation of Δ*mntP* was assessed using strains JK895, EF737, and EF738. Complementation of Δ*mntP* Δ*yiiP* was assessed using JK895, EF739, EF740, and EF741. Complementation of Δ*mntP* Δ*yiiP* with the YiiP D45H mutant construct pEF108 was assessed using strains JK895, EF739, EF741, and EF773. Growth assays were carried out as described above except MnSO_4_ was added at both 0.5 mM and 0.25 mM final concentration.

Strains used to determine zinc sensitivity in response to YiiP and MntP expression were EF564, EF745, EF746, and EF762. Zinc sensitivity of manganese transporter mutants was assessed using strains JK237, EF561, EF635, and EF657. Zinc sensitivity assays were carried out as described above but the LB was supplemented with 0.125 mM or 0.5 mM ZnSO_4_ respectively.

### Metal sensitivity spot assays.

Strains were grown overnight in LB, diluted 1:1000 in 5 mL fresh medium then grown with shaking at 37°C for 3 h to OD_600_ = 0.3. Cultures were serially diluted 10-fold in PBS, then 3 μL were spotted onto LB agar plates with or without metal sulfate supplementation. Plates were grown 14–16 h at 37°C prior to imaging. Spot assays utilized the same strains as growth curve assays. Complementation of manganese sensitivity by expression of FLAG-tagged YiiP proteins was assessed using strains EF564, EF747, EF829, and EF830.

### Metal content analyses.

Inductively coupled plasma-mass spectrometry (ICP-MS) analyses were conducted using strain variants that were also flagellar mutants (EF610, EF697, EF698, EF701) to increase pelleting efficiency as in previous studies (14, 28). Overnight cultures were diluted 1:1000 into 100 mL fresh LB medium and then grown to OD_600_ ∼ 1. Cultures were divided into 5 mL aliquots in 18 × 150 mm glass tubes, treated with 2 mM diethylamine NONOate (DEANO) and returned to shaking at 37°C. At 0, 5, 15, 30, 45, and 60 min posttreatment, 4.5 ml of culture was pelleted by centrifugation then washed twice with ultrapure water. Pellets were resuspended in analytical grade nitric acid, boiled, then diluted 1:10 with ultrapure water for analysis on an Agilent 8900x QQQ ICP-MS. Bacterial numbers, defined as CFU, were enumerated at each time point for calculation of relative metal concentrations. Statistical significance was determined by two-tailed *t* test in Microsoft Excel.

### Gene expression analysis.

Primer sequences used for expression analysis are listed in Table 2. For expression under metal chelation, wild-type *S*. Typhimurium was grown overnight, diluted 1:1000 in 2 × 5 mL fresh LB medium, and grown to OD_600_ ∼ 1. Ethylenediaminetetraacetic acid (EDTA) at a concentration of 3 mM was added to one of the cultures for 20 min. 1.5 ml of each culture was pelleted by centrifugation then resuspended in 800 μL TRIzol for RNA isolation.

For analysis of expression in response to metal supplementation, overnight LB cultures were diluted 1:1000 into 25 mL modified Tris minimal medium (50 mM Tris, 80 mM NaCl, 2 mM KCl, 5 mM NH_4_SO_4_, 1.65 mM Na_2_SO_4_·10H_2_O, 1 mM MgSO_4_·6H_2_O, 0.3 mM CaCl_2_, 1.6 mM Na_2_HPO_4_, 0.2% glucose, 3 g.L^−1^ casamino acids) and grown to OD_600_ ∼ 0.5. The culture was subdivided into 4 × 5 mL cultures in 18 × 150 mm glass tubes. One was left untreated and the others were supplemented with 0.5 mM FeSO_4_ plus 1 mM ascorbate, 0.5 mM MnSO_4_, or 0.5 mM ZnSO_4_. After 20 min, 3 mL of culture was pelleted by centrifugation and pellets were resuspended in 800 μL TRIzol for RNA isolation.

To monitor expression in response to nitrosative stress, wild-type *S*. Typhimurium was grown overnight, diluted 1:1000 in 100 mL fresh LB medium, and grown to OD_600_ ∼ 1. The culture was subdivided into 5 duplicate pairs of 5 mL cultures in 18 × 150 mm glass tubes. One set was treated with 2 mM DEANO and one set left untreated. Cultures were returned to shaking at 37°C. At 5, 15, 30, 45, and 60 min posttreatment, 1.5 ml of treated and untreated culture was pelleted by centrifugation then resuspended in 800 μL TRIzol for RNA isolation.

To determine whether supplementation of growth medium with manganese disrupts zinc homeostasis and expression of zinc-regulated zinc transporters, JK237 and EF657 *S*. Typhimurium were grown overnight, diluted 1:1000 in 2 × 5 mL fresh LB medium, and grown to OD_600_ ∼ 0.5. One culture was left untreated and the other was supplemented with 0.5 mM MnSO_4_ before cultures were returned to shaking at 37°C. At 20 and 60 min posttreatment, 1.5 mL of treated and untreated culture was pelleted by centrifugation then resuspended in 800 μL TRIzol for RNA isolation.

For all analyses, RNA and cDNA were prepared as described previously (64). Quantitative PCR (qPCR) was carried out using SYBR green on a Bio-Rad CFX96 real-time system with *rpoD* as the internal control for normalization. Fold change values (treated/untreated) were log_2_ transformed prior to plotting. Statistical significance was determined by one-sample *t* test compared to a hypothetical means of 1 or −1 using GraphPad Prism.

### Western blotting.

Strains EF829 and EF830 were grown as for spot assays, then 3 mL of culture was pelleted, resuspended in 100 μL PBS, and diluted 1:1 in 2× Laemmli sample buffer (Bio-Rad) with DTT (100 mM final). Samples were incubated 60 min at 55°C before 7.5 μL was loaded on a 4–20% Tris-glycine gel for separation in Tris-glycine-SDS buffer (Bio-Rad). Separated proteins were transferred to nitrocellulose membranes, which were blocked with EveryBlot Blocking Buffer (Bio-Rad) then probed with 1:000 monoclonal anti-FLAG M2-peroxidase (HRP) antibody (Millipore Sigma). Blots were visualized using an ECL Western blotting analysis system (Amersham) on an ImageQuant LAS 4000 imaging system (GE Healthcare) that captures both chemiluminescent and visible images. The visible image of the protein size standards was aligned with the chemiluminescent image of the same blot.
